# Exploring lifelong learning organization with Endogenous and Exogenous concept: A Case Study of Japan and Taiwan

**DOI:** 10.1093/geroni/igaf122.2944

**Published:** 2025-12-31

**Authors:** Yunann Lin, Huichuan Wei, Wang Chih-Liang

**Affiliations:** National Chung Cheng University, Minxiong, Chiayi, Taiwan (Republic of China); National Chung Cheng University, Minxiong, Chiayi, Taiwan (Republic of China); National Cheng Kung University, Tainan City, Tainan, Taiwan (Republic of China)

## Abstract

In response to the VUCA (Volatility, Uncertainty, Complexity, Ambiguity) era, lifelong learning organizations must undergo transformative changes as societies emphasize mutual recognition and self-discovery through interaction. This study explores Taiwan’s transition by analyzing Japan’s multi-layered community-based lifelong learning organizations. The Tamago project in Kashiwa City fosters lifelong learning through community cafés and diverse activities, including educational programs, board game creation, festivals, and holiday events. The Huwei Active Aging Learning Center (AALC), established by Taiwan’s Ministry of Education, offers diverse courses focusing on intergenerational interaction, health promotion, and liberal arts. This study employs Bereday’s comparative method through interviews, on-site visits, and literature review to analyze the organizational structure, mission, curriculum design, and participant demographics of these two lifelong learning organizations. The analysis reveals distinct differences: Kashiwa operates as an autonomous, resident-managed organization, while Huwei AALC functions as a government-commissioned institution with certified instructors and local managers. Kashiwa emphasizes community problem-solving and collaborative teaching, facilitating intergenerational skill-sharing, whereas Huwei follows a curriculum-based approach targeting older adults over 55. Despite similarities in course content and formation, the two models reflect fundamentally different developmental approaches—endogenous (Kashiwa) versus exogenous (Huwei). Both successfully strengthen localized small-scale social interactions, fostering environments for social connection, self-actualization, and self-identity. Taiwan could benefit from Kashiwa’s intergenerational approach to enhance intrinsic motivation among older adults. Future implications include optimizing the learning environment to enhance civic literacy and redefining government roles to view older adults as valuable societal resources

